# Introduction of mammalian seed predators and the loss of an endemic flightless bird impair seed dispersal of the New Zealand tree *Elaeocarpus dentatus*

**DOI:** 10.1002/ece3.4157

**Published:** 2018-05-08

**Authors:** Joanna K. Carpenter, Dave Kelly, Elena Moltchanova, Colin F. J. O'Donnell

**Affiliations:** ^1^ Centre for Integrative Ecology School of Biological Sciences University of Canterbury Christchurch New Zealand; ^2^ Department of Math and Statistics University of Canterbury Christchurch New Zealand; ^3^ Department of Conservation Biodiversity Group Christchurch New Zealand

**Keywords:** frugivore decline, hinau, invasive mammal, seed predation, weka

## Abstract

Understanding the mutualistic services provided by species is critical when considering both the consequences of their loss or the benefits of their reintroduction. Like many other Pacific islands, New Zealand seed dispersal networks have been changed by both significant losses of large frugivorous birds and the introduction of invasive mammals. These changes are particularly concerning when important dispersers remain unidentified. We tested the impact of frugivore declines and invasive seed predators on seed dispersal for an endemic tree, hinau *Elaeocarpus dentatus,* by comparing seed dispersal and predation rates on the mainland of New Zealand with offshore sanctuary islands with higher bird and lower mammal numbers. We used cameras and seed traps to measure predation and dispersal from the ground and canopy, respectively. We found that canopy fruit handling rates (an index of dispersal quantity) were poor even on island sanctuaries (only 14% of seeds captured below parent trees on islands had passed through a bird), which suggests that hinau may be adapted for ground‐based dispersal by flightless birds. Ground‐based dispersal of hinau was low on the New Zealand mainland compared to sanctuary islands (4% of seeds dispersed on the mainland vs. 76% dispersed on islands), due to low frugivore numbers. A flightless endemic rail (*Gallirallus australis*) conducted the majority of ground‐based fruit removal on islands. Despite being threatened, this rail is controversial in restoration projects because of its predatory impacts on native fauna. Our study demonstrates the importance of testing which species perform important mutualistic services, rather than simply relying on logical assumptions.

## 1 INTRODUCTION

Drivers of global environmental change such as habitat loss, illegal harvesting, and biological invasions have had negative impacts on frugivorous, seed‐dispersing species, sparking concern for the functioning of seed dispersal networks (Sekercioglu, Daily, & Ehrlich, 2004). Frugivorous animals influence the survival, community dynamics (Wright et al., 2000), and spatial and genetic patterns of plants (Levine & Murrell, 2003; Nathan & Muller‐Landau, 2000), so frugivore declines can have significant cascading effects, although these consequences are frequently masked by the long life span of perennial plants (McConkey et al., 2012). Many ecosystems are already suffering from low biodiversity following hundreds of years of human impacts, which further exacerbates the effects of recent disperser declines (Corlett, 2007; O'Farrill, Galetti, & Campos‐Arceiz, 2013). Although some cascading effects such as impaired plant recruitment have been documented (e.g., Christian, 2001; Rogers et al., 2017; Wotton & Kelly, 2011), the effects of frugivore losses on their mutualistic partners are complex and still poorly understood. This is particularly true when unexpected animals are acting as seed dispersers (Calviño‐Cancela, 2002; Young, Kelly, & Nelson, 2012) or where unusual dispersal mechanisms occur that may have been overlooked (e.g., Wallace, Howell, & Lee, 2008).

In addition to declines in frugivores, ecosystems worldwide have suffered from biological invasions. Invading species have the potential to either directly alter seed dispersal networks, by the establishment of novel interactions with native biota, or indirectly alter seed dispersal networks, by affecting the abundance, behavior, or distribution of native biota (McConkey et al., 2012). Invasive mammals such as rodents are particularly pervasive and problematic, with ship rats (*Rattus rattus*) having invaded over 80% of the world's island groups (Towns, 2009). Rodents have the capacity to damage seed dispersal interactions by destroying or depredating (we will use these two terms synonymously) seeds (Pender, Shiels, Bialic‐Murphy, & Mosher, 2013; Shiels & Drake, 2015) and preying upon native frugivores (Towns, Atkinson, & Daugherty, 2006). While the impacts of exotic mammals on populations of frugivores have been well established (Doherty, Glen, Nimmo, Ritchie, & Dickman, 2016), the synergistic effects of exotic mammalian seed predators and declines in native dispersers are largely unknown (McConkey et al., 2012; but see Wotton & Kelly, 2011), despite their ubiquity.

New Zealand unfortunately offers an ideal opportunity to test the effects of frugivore declines and exotic mammals on seed dispersal services. The archipelago's 80 million year isolation from other landmasses has created an unusual suite of frugivores, dominated by birds and lizards and almost entirely devoid of mammals (Kelly et al., 2010; Wotton, Drake, Powlesland, & Ladley, 2016). Since the arrival of humans in ca. 1280 (Wilmshurst, Anderson, Higham, & Worthy, 2008), almost half (41%) of New Zealand's endemic avifauna has gone extinct, including many frugivores (Innes, Kelly, Overton, & Gillies, 2010). These considerable losses are partly due to the introduction of mammalian predators, including three species of rat (Polynesian rat *Rattus exulans*, Norway rat *Rattus norvegicus,* and ship rat *Rattus rattus*), mice *Mus musculus*, cats *Felis catus*, mustelids (Mustelidae), and brushtail possums *Trichosurus vulpecula* (Towns et al., 2006). These mammals affect seed dispersal both indirectly, by preying upon frugivore populations (e.g., Clout, Karl, Pierce, & Robertson, 1995; Innes et al., 2010; Starling‐Windhof, Massaro, & Briskie, 2011), and directly through seed and flower predation (Beveridge, 1964; Campbell & Atkinson, 2002). For example, Wotton and Kelly (2011) demonstrated that the synergistic effects of frugivore loss and mammalian seed predation reduced recruitment of two large‐seeded New Zealand trees by >92%.

**Figure 1:**
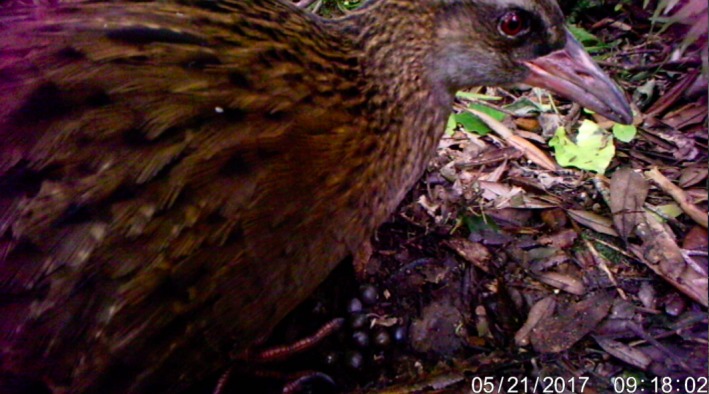
Still capture from trail camera footage, showing a weka *Gallirallus australis* approaching a depot of hinau *Elaeocarpus dentatus* fruits on Blumine Island

So far, investigations into the functioning of New Zealand's seed dispersal networks have focused on volant frugivorous birds (Kelly et al., 2010). However, flightless frugivores were a substantial part of New Zealand's historic avifauna, and the role that these species play in seed dispersal is still unclear. Understanding whether flightless birds are significant seed dispersers in New Zealand may also provide information on whether removal of fruits by ground‐based birds is an important mechanism on other oceanic islands where flightlessness is common (e.g., Polynesia). Like Polynesia (Olson & James, 1991; Steadman, 1995), a large proportion of the birds in this guild have gone extinct in New Zealand (66%: Atkinson & Millener, 1991; Tennyson, 2009). One species that remains is the endemic weka (*Gallirallus australis*; Figure 1), a charismatic flightless rail that has become severely range restricted due to mammalian predation and possible climate‐related starvation (Beauchamp, Butler, & King, 1999). Their large gape and frequent consumption of fruit suggests they may be significant seed dispersers (Carroll, 1963; Coleman, Warburton, & Green, 1983), but their predatory impacts on other native fauna have led to them becoming regarded negatively by conservationists (Miskelly & Beauchamp, 2004). Their predatory behavior has resulted in the removal of weka from at least eleven islands where humans had introduced them (Miskelly & Beauchamp, 2004), and even from some islands where they occurred naturally (e.g., Anchor Island, Fiordland).

Ground collection of fruit by flightless birds such as weka is likely to have been an important dispersal mechanism for many plant species, particularly those with larger fruits (Lee, Clout, Robertson, & Bastow Wilson, 1991; Thorsen, Seddon, & Dickinson, 2011). For example, in Australia, cassowaries (*Casuarius* spp.) and emus (*Dromaius novaehollandiae*) remove a significant proportion of seeds from the ground (Bradford & Westcott, 2010; Calviño‐cancela et al., 2006). Lord (2002) speculated that seeds that were adapted for dispersal by flightless birds should fall to the ground when ripe and be conspicuous on the forest floor. One species that meets these criteria is hinau (*Elaeocarpus dentatus*: Elaeocarpaceae), an endemic forest tree with large, shiny dark brown fruits that feature a very thick endocarp and drop to the ground when ripe (Lord, 2002). Hinau currently appears to have very low seed dispersal rates from the canopy (10%–28% of seeds captured beneath parent trees have passed through a bird; Carpenter, Kelly, Clout, Karl, & Ladley, 2017), but it is unclear whether these low rates are due to low local numbers of volant frugivores, or because its seeds are adapted for ground removal by flightless frugivores. The only extant frugivores recorded consuming hinau fruits are volant kereru (*Hemiphaga novaeseelandiae*) and kokako (*Callaeas wilsoni*), and flightless weka and brown kiwi (*Apteryx mantelli*) (Clout & Hay, 1989; Kelly et al., 2010), three of which (kokako, brown kiwi, and weka) are severely range restricted. Additionally, rats and feral pigs (*Sus scrofa*) have been recorded destroying hinau seeds (Beveridge, 1964; Daniel, 1973), and brushtail possums commonly eat the flesh from the fruits and drop the seeds undispersed below the parent tree (Cowan & Waddington, 1990). Consequently, hinau could be suffering from dispersal limitation across most of the mainland where mammalian seed predators are common and few of its dispersers occur.

Conservation efforts in New Zealand have eradicated exotic mammals from many offshore islands and fenced sanctuaries (Parkes, Byrom, & Edge, 2017; Towns & Broome, 2003), bolstering frugivore populations (Graham & Veitch, 2002; Graham, Veitch, Aguilar, & Galbraith, 2013; Iles & Kelly, 2014; Murphy & Kelly, 2001) and restoring a more intact ecosystem (Saunders & Norton, 2001; Tanentzap & Lloyd, 2017). For example, endemic bellbird (*Anthornis melanura*) densities on the Poor Knights Islands (a sanctuary that has never been invaded by exotic mammals) are 54 times greater than average densities on the New Zealand mainland (North and South Islands) (Bartle & Sagar, 1987). These islands offer the opportunity for testing the influence of native dispersers and exotic mammals on seed dispersal rates through comparisons between island avifaunas with high bird densities and bird species of restricted distributions (Graham et al., 2013; Iles, 2012) and depauperate mainland sites. We used replicated, paired mainland and island sanctuary sites to assess whether hinau is dispersal limited on the mainland and whether it appears adapted for dispersal by flightless birds such as weka. We also used these sites to assess seed predation rates by exotic mammals. Specifically, we aimed to answer the following questions:

1. What proportion of hinau fruits are handled by frugivores in the canopy (the percentage of fruits captured below trees that have passed through a bird; an index of dispersal quantity), and does this proportion vary with abundance of volant frugivores?
2. What proportion of hinau fruits which reach the ground is dispersed from there, and does that vary between predator‐free island sanctuaries and mainland sites?
3. What species of frugivore remove hinau fruits from the ground, and how important among these are weka?
4. What levels of seed predation does hinau experience, and are seed predation rates lower on predator‐free islands than on the mainland?

## 2 METHODS

### 2.1 Study species

**Figure 2:**
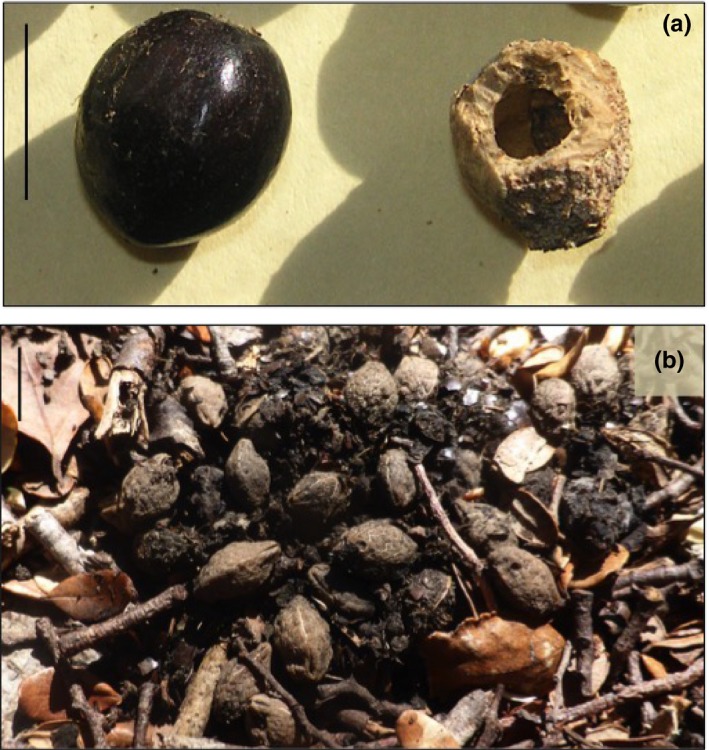
(a) Photograph depicting a whole, ripe hinau fruit on the left, and a rat destroyed hinau seed on the right. (b) Photograph of weka feces on D'Urville Island containing >13 whole hinau seeds, courtesy of Geoff Walls. Scale bars are 10 mm

Hinau is a lowland forest tree that occurs across the North Island and the West Coast of the South Island. Its fruits are oval purple‐brown drupes measuring 9.2 mm diameter on average (Kelly et al., 2010), with a high percentage of flesh (the mesocarp, 25% by mass) and a relatively low water content (66%; Williams, 1982). The seed is protected inside a hard, thick seed coat (the endocarp) so that rodents can only destroy the ripe seeds by gnawing through the seed coat (Figure 2a; Beveridge, 1964; Daniel, 1973), although kaka *Nestor meridionalis* (an endemic parrot) split the seed coat while it is still green and consume the developing seed (Moorhouse, 1997). Fruit crop size is variable from year to year, ranging from <1,000 to more than 30,000 fruits per tree (Cowan & Waddington, 1990). Hinau's seed fall coefficient of variation is 0.83, which defines it as a moderately masting species when compared to other New Zealand plants (Kelly & Sork, 2002; Kelly et al., 2013; Webb & Kelly, 1993). Fruits ripen and fall between March and September.

### 2.2 Sites

**Figure 3:**
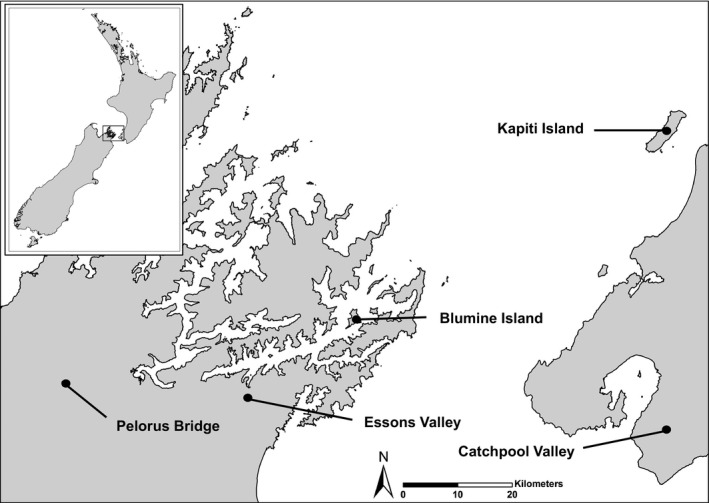
Map showing the paired, mainland–island sites, and their location in wider New Zealand (inset). Blumine Island and Essons Valley are the South Island pair, while Kapiti Island and Catchpool Valley are the North Island pair. Fruit handling indices from seed traps at Pelorus Bridge were used as a surrogate for Essons Valley, where seed traps were not established

Monitoring occurred at two island/mainland pairs located in central New Zealand: one pair in the upper South Island (Blumine Island/Oruawairua −41°17′47 S, 174°24′10 E, and Essons Valley 41°30′46 S, 174°00′94 E) and one pair in the lower North Island (Kapiti Island −40°85′18 S, 174°91′41 E, and Catchpool Valley −41°35′10 S, 174°92′57 E) (Figure 3). Kapiti Island is approximately 54 km from Catchpool Valley, and Blumine Island is approximately 23 km from Essons Valley. The two islands have high levels of native frugivorous birds such as weka, kereru, tui *Prosthemadera novaeseelandiae*, and bellbirds, as well as frugivores now rare or absent from the mainland (tieke *Philesturnus carunculatus*, kiwi *Apteryx* spp., kaka, and kakariki *Cyanoramphus* spp.) (Robertson, Hyvőnen, Fraser, & Pickard, 2007). The mainland sites contain a suite of introduced mammalian species including brushtail possums, ship rats, Norway rats, house mice, and feral pigs, which are absent from the island sites (King, 2005). As a result, they have lower numbers of native frugivorous birds such as kereru, tui, and bellbirds (Iles & Kelly, 2014; Murphy & Kelly, 2001; Robertson et al., 2007). Essons Valley has low numbers of weka (Pers. Obs.) but Catchpool has none (Robertson et al., 2007).

### 2.3 Indices of dispersal and seed predation from the canopy

Seed traps were established beneath the canopies of ten hinau trees per site on Kapiti Island, Blumine, and Catchpool Valley to obtain fruit handling and seed predation indices from the canopy. Fruit handling indices were comprised of the proportion of seeds captured that had passed through a bird, and seed predation indices were comprised of the proportion of seeds captured that had been destroyed. Each seed trap was comprised of a 41 cm × 29 cm × 6.2 cm plastic seed raising tray, covered with plastic mesh to discourage fruit removal from the traps, and pegged securely to the ground. Two traps were set up beneath each tree, giving a catching area of 0.24^ ^m^2^ per tree. Seed traps were established in March or April 2017 and were checked monthly until September 2017 (the end of the hinau fruiting season). Fruits were classed as either passed through a frugivore (fruit skin removed but no visible chew damage), preyed on by native parrots (endocarp cleaved in half, destroying the seed inside), chewed by possums (exocarp and mesocarp removed with chew marks), or intact whole fruits found under parent trees (both ripe and unripe). Fruits that had passed through a frugivore were distinguished by their slippery texture, with some mesocarp remaining on the seed (Carpenter et al., 2017). Further seed trap data were obtained from the Department of Conservation's national seed rain monitoring network, which gave fruit handling indices from an additional mainland site at Pelorus Bridge (Marlborough), about 35 km west of Essons Valley. The fruit handling data obtained from seed traps at this site were used a surrogate for Essons Valley, where no seed traps were established. Pelorus Bridge used elevated conical seed traps with a catching area of 0.28 m^2^; see Carpenter et al. (2017) for a description. Kereru, bellbirds, and tui occurred in low numbers at this site (Carpenter et al., 2017; Robertson et al., 2007). Rodents and possums were also present.

### 2.4 Bird visitation rates to the canopy

Between April and June, eight trail cameras were each trained on a fruiting branch in the canopy of a hinau tree across three of the sites (one on Kapiti Island, three at Catchpool Valley, and four on Blumine Island). As these trees needed to have suitable low‐hanging branches, different trees were selected from the trees that had seed traps and ground cameras below them. Cameras were mounted on the top of 5.5 m telescopic poles, secured with guy lines and pegs. Cameras were set on motion detect photographic mode to obtain images of volant bird visitation rates over 2 weeks at each site. The five cameras on Blumine Island were left for an additional 3 weeks monitoring to maximize the chance of recording volant frugivores.

### 2.5 Fruit removal and destruction rates on the ground

Motion‐triggered video camera traps were used to positively identify species that dispersed or destroyed deposits of hinau seeds that we placed on the forest floor. Seeds that were removed by rodents or pigs were classed as depredated. Ship rats, Norway rats, Polynesian rats, and mice remove seeds for consumption at safe, sheltered sites, but they do not display scatter‐hoarding behavior (burying seeds in widely spaced caches), and hinau seeds are too large for them to swallow and disperse intact. Instead, rodents destroy hinau seeds by gnawing through the seed coat (Beveridge, 1964; Daniel, 1973; Grant‐Hoffman & Barboza, 2010). Pigs eat and crush whole hinau fruits, with pig guts containing large quantities of destroyed hinau seeds (Beveridge, 1964). Fruits that were removed by weka or kereru were classed as dispersed as these species swallow the fruits and defecate the seeds intact (Figure 2b; Geoff Walls personal communication; Beauchamp, 1987; Kelly et al., 2010).

One trail camera (either a LTL Acorn 5310A Wide Angle Trail & Security Camera, KeepGuard KG690NV 8MP Wildlife Camera, or Moultrie Game Spy M‐990i Gen 2 10.0 MP Camera) was placed 50–200 cm in front of a depot of ripe hinau fruit beneath the canopy of each of ten fruiting hinau trees per site. These were the same trees that had seed traps below them at Kapiti Island, Blumine Island, and Catchpool Valley. Cameras were mounted about 1 m above the ground. Ten ripe fallen fruits were placed in a small depression on the ground cleared of leaf litter and debris (Moles & Drake, 1999). For trees that did not have enough fallen fruit beneath them to create a depot of monitored fruits, we used fruits from nearby trees. Where mammals were present, fruits were handled using latex gloves rinsed in water to avoid affecting disperser behavior with human scent (Wenny, 2002). The number of fruits dispersed or preyed upon was recorded after 2, 9, and 14 days, and then, the cameras were removed. Hinau fruits remain fresh for many weeks on the ground (Pers. Obs.) and were still in excellent condition when monitoring finished. Camera footage was used to identify the animal species that interacted with fruits. Monitoring occurred between April and June 2017 (the peak of the hinau fruiting season).

### 2.6 Analysis

We used binomial generalized linear mixed‐effects models (GLMMs) in a classical framework to assess whether fruit handling rates from the canopy differed between islands and the mainland, and whether fruit removal by dispersers from the ground differed between island and mainland sites. For the fruit handling rates from the canopy model, the proportion of fruits per trap per year (March – September) that had passed through a bird was the response variable, site status (mainland or island) was the fixed effect, and site was the random effect. For the ground dispersal model, proportion of all fruit in the depot removed by legitimate dispersers (weka, kereru) was the response variable, site status was the fixed effect, and site was a random effect. We corrected for overdispersion in this model using an observation level random effect (Browne, Subramanian, Jones, & Goldstein, 2005).

In order to assess the importance of various dispersers and seed predators, we used Bayesian statistics (Ellison, 2004) to test for differences among ground‐based frugivores in the mean percentage of hinau fruit they removed. We were interested in determining the probability that a seed placed onto the forest floor would be removed by each of the species present at a site. Frugivore species were only included as present at a site if they were detected on the ground by a camera and had removed a fruit at one of the sites. Using these criteria, Blumine had weka and kereru, Kapiti had only weka, Essons had weka, rats, mice, and pigs, and Catchpool had rats and mice. We fitted a mixed‐effects logistic multinomial regression model using the deviance information criterion (DIC) to select the best statistical model (Spiegelhalter, Best, Carlin, & Van Der Linde, 2002). Our response variable was a vector consisting of the number of seeds removed by each frugivore type, with site and camera ID included as random effects. We used the statistical software package WinBUGS (Spiegelhalter et al., 2002) for our analysis and the software R (R Development Core Team, 2010) for additional posterior probabilities.

## 3 RESULTS

### 3.1 Hinau dispersal

**Figure 4:**
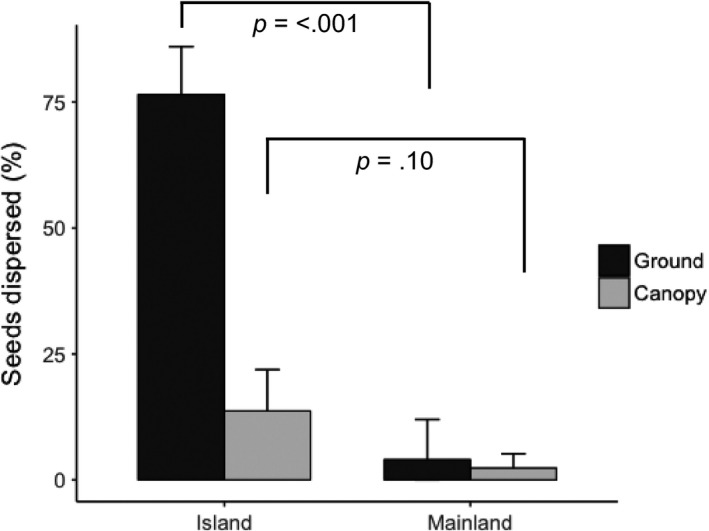
Mean percentage of seeds consumed by dispersers (ground seed data from camera traps) or passed through a bird (canopy seed data from seed traps) for island (*n* = 2 sites) and mainland sites (*n* = 2 sites). Error bars are bootstrapped 95% confidence intervals

Seed trap data from all sites showed that there was no significant difference between canopy dispersal rates (proportion of seeds in seed traps that had been through a frugivore) on the mainland (Catchpool Valley and Pelorus) compared to the islands (Blumine Island and Kapiti Island; *Z* = −1.642, *p* = .10). Low levels of dispersal occurred at all four sites (mean of 13.7% canopy seeds dispersed for islands [41 of 278 captured seeds] and 2% on the mainland [14 of 514 captured seeds]; Figure 4). Thus, canopy dispersal of hinau fruit was uncommon even on islands with high abundances of endemic birds.

The four canopy cameras on Blumine Island provided between 6 and 36 days of usable footage each. The camera on Kapiti Island provided 14 days of footage, and the three cameras at Catchpool each provided 14 days of footage. This added up to 42 days footage from the mainland site and 124 days footage from the two island sanctuary sites. No frugivorous birds visited the tree canopies at Catchpool over the 2 weeks of monitoring, although possums were recorded. Five visits from kereru and two from tui were recorded on Blumine. A single visit by a kaka was recorded on Kapiti Island. As the cameras were set to take photographs rather than video footage, fruit consumption by each individual bird was not assessed. There were not enough data to analyze canopy visitations, but these preliminary results show that few birds visited hinau canopies even on islands with high numbers of birds.

**Figure 5:**
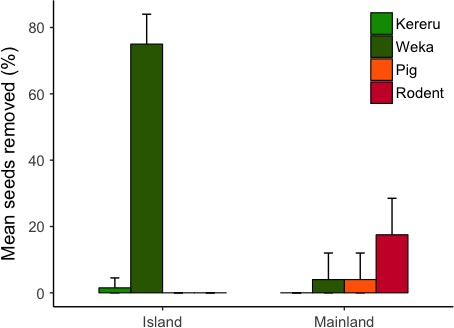
Mean percentage of ground fruits removed by each species across island and mainland sites. Green colors denote endemic seed dispersers, and red colors denote exotic seed predators. Error bars are bootstrapped 95% confidence intervals

Hinau seeds on the ground were far more likely to be dispersed on islands (76.5% of seeds dispersed; 153 of 200 monitored seeds), than at the two mainland sites (4%; eight of 200 monitored seeds from Catchpool Valley and Essons Valley). Site status was a significant effect in our GLMM (*Z* = −5.489, *p* = <.001; Figure 4). Weka and kereru were the only two dispersers recorded consuming fruits from ground depots. Blackbirds (*Turdus merula*), song thrushes (*Turdus philomelos*), tieke, robins (*Petroica* spp.), and little spotted kiwi (*Apteryx owenii*) were all detected by cameras but were not seen to consume any fruit. Our Bayesian probability analysis found that weka were the most likely species to consume hinau fruits off the ground on island sanctuaries (Figure 5; likelihood of weka removing a fruit rather than other species on islands >0.9999). Weka feces filled with hinau seeds were a common sight on Blumine Island in particular. In summary, we recorded high proportions of hinau seeds on the ground being dispersed (predominantly by weka) on islands, with much lower dispersal levels at mainland sites.

### 3.2 Hinau seed predation

No seeds on the ground were destroyed at the two island sites (0 of 200 monitored seeds), but 21.5% seeds (43 of 200 monitored seeds) were removed by rodents or pigs (and therefore assumed to be destroyed) at the two mainland sites (Catchpool Valley and Essons Valley). Rodents were the most likely taxon to remove a seed from the ground at the mainland sites (Figure 5; probability of a rodent removing a fruit compared to other species present at the sites = 0.9 and 0.99 for Catchpool Valley and Essons Valley, respectively). Possums had chewed an average of 55% of fruits on the ground (110 of 200 monitored seeds) at the two mainland sites but did not destroy or remove any seeds. However, possum handling of fruits might have a small negative effect by reducing fruit attractiveness to legitimate dispersers (see Discussion).

Data from the seed traps showed that over the entire fruiting season, endemic parrots destroyed 32.5% of the seeds from the canopy at the two island sites (90.5 of 278 captured seeds). No seeds were destroyed from the canopy at Catchpool Valley, although possums in the canopy had chewed 91.7% (364 of 397 captured seeds) of seeds captured in seed traps at this site. In summary, exotic seed predators removed and likely destroyed 21.5% of seeds on the ground at the mainland sites in 2 weeks, while no seeds were destroyed on the ground on island sanctuaries. However, endemic parrots on islands destroyed 32.5% of seeds from the canopy over the entire fruiting season (~6 months).

### 3.3 Possible combined impact of seed predation and dispersal

**Figure 6:**
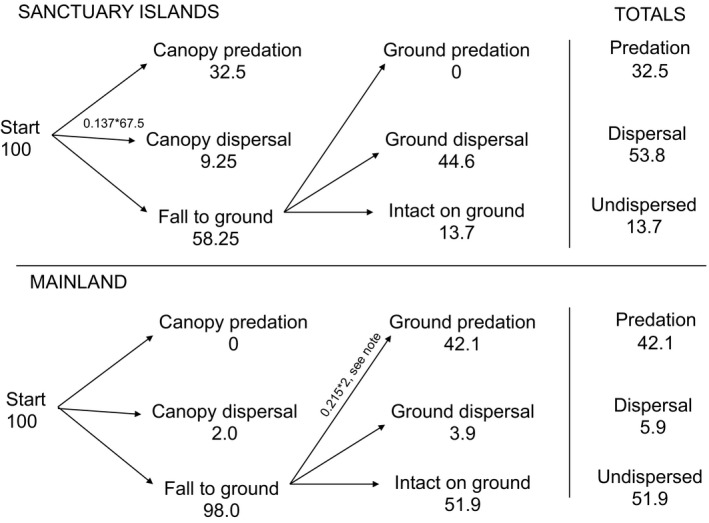
Flowchart showing one possible integration of the effects of seed predation and dispersal on sanctuary islands and the mainland over an entire fruiting season. The chart starts with 100 seeds at each location type (sanctuary islands vs. mainland) and plots their sequential fates, using percentages of seed predation and dispersal obtained from camera traps and seed traps. This integrative approach uses figures obtained from a range of different methods and is therefore speculative. Note: For the purposes of this composite summary, we have assumed the percentage of seed predation by exotic mammals that we recorded over 2 weeks (21.5%) would double if we had measured for an entire 6‐month fruiting season (see Discussion). Therefore, we have assumed a predation rate of 43% for this composite summary

Using the figures above, we present one possible integration of the effects of hinau seed predation and dispersal on the mainland compared to island sanctuaries over an entire fruiting season. It is important to note that this integrative approach uses figures obtained from a range of different methods and is therefore speculative. Our composite summary (Figure 6) follows the sequential fate of 100 seeds on both sanctuary islands and the mainland, using the percentages of seeds that were dispersed or destroyed at each stage (canopy and ground). The results of this summary demonstrate that on islands, 32.5% of hinau seeds are destroyed, 53.8% are dispersed, and 13.7% are undispersed. On the mainland, 42.1% of seeds are destroyed, 5.9% are dispersed, and 51.9% are undispersed. The key finding is that on sanctuary islands most undestroyed hinau seeds are dispersed, while on the mainland, they remain undispersed beneath the parent tree.

We made several assumptions when calculating this possible integration of seed predation and dispersal. As mammalian predation from the ground was only measured for 2 weeks, we assumed that this rate of predation would double if we had measured for the entire 6‐month fruiting season (see discussion), so we have used a mammalian predation rate of 43%. While this rate is very speculative, it correlates well with the rate of mammalian predation on hinau seeds recorded by other studies (Overdyck, Clarkson, Laughlin, & Gemmill, 2013). We also assumed that parrot seed predation was on green fruit only (as the endocarp of ripe fruit is too hard for parrots to destroy; Moorhouse, 1997), therefore removing fruits from the potential dispersal pool before they could be dispersed. Finally, for the purpose of this summary, we assumed that fruits that were dispersed from the canopy were not vulnerable to ground predation (as the flesh from the fruit is removed, making the fruits unattractive to seed predators).

## 4 DISCUSSION

We found that hinau had significantly less dispersal from the ground at mainland sites where native frugivores have declined or gone locally extinct compared to island sanctuaries that more closely approximate prehuman frugivore communities. On sanctuary islands where weka were abundant, the majority of seeds that fell to the ground were consumed and dispersed by weka, while on the mainland, the majority of seeds on the ground were chewed by possums and left in situ. Most of the remaining seeds on the ground at mainland sites were removed, and likely destroyed, by exotic seed predators such as rodents and pigs. As we expected, dispersal rates from the canopy were poor at both island and mainland sites, possibly because hinau fruit falls to the ground when ripe rather than being retained in the canopy. This is consistent with kereru being observed consuming fruit both in the canopy and from the ground. All this suggests that hinau is adapted for dispersal by flightless birds (discussed further below).

Several other studies have demonstrated that frugivore declines caused by invasive species can have cascading effects on seed dispersal services. In New Zealand, *Pittosporum crassifolium* experienced poor seed dispersal (20% of seeds removed by birds) at mainland sites compared to sanctuary island Tiritiri Matangi Island (94% removal) (Anderson, Kelly, Robertson, Ladley, & Innes, 2006), and nikau *Rhopalostylis sapida* and *Fuchsia excorticata* also have impaired dispersal on the mainland compared to Kapiti Island (McNutt, 1998). Tawa *Beilschmiedia tawa* also appears to suffer from extremely low dispersal at some mainland sites (<10% of seeds captured below parent canopies had passed through a bird at three North Island sites; Silberbauer, 2013), although eight other studies on seed dispersal quantity in mainland New Zealand have found adequate dispersal rates (Kelly et al., 2010; Pegman, Perry, & Clout, 2017). On the Balearic Islands, introduced carnivorous mammals have indirectly lowered seedling recruitment of a perennial shrub by driving its mutualistic partner extinct (Traveset & Riera, 2005), while on Guam, the near‐total loss of frugivorous birds caused by the exotic brown tree snake (*Boiga irregularis*) may have caused a 61%–92% decline in seedling recruitment for two plant species (Rogers et al., 2017) and a reduction in seedlings of all tree species reaching canopy gaps away from parent trees (Wandrag, Dunham, Duncan, & Rogers, 2017). Whether the reduced dispersal we have recorded results in lowered recruitment for hinau depends on hinau's reliance on avian dispersal for improved germination (e.g., by gut passage, see Robertson, Trass, Ladley, & Kelly, 2006) and escape from disproportionate density‐ and distance‐dependent mortality beneath parent canopies (i.e., Janzen–Connell effects, see Comita et al., 2014). A New Zealand study on two other large‐fruited native trees (*Corynocarpus laevigatus* and *Beilschmiedia tarairi*) found better recruitment to the 2‐year‐old seedling stage away from parents of both species, suggesting that frugivore declines would reduce regeneration (Wotton & Kelly, 2011). Carpenter, Wood, Wilmshurst, and Kelly (2018) demonstrated that simulated avian gut passage may increase the germinability of hinau compared to whole fruits, so the results we have shown could have flow‐on effects to recruitment, but further research is needed to examine hinau's susceptibility to Janzen–Connell effects.

### 4.1 The importance of weka

Weka have disappeared from most regions of the North and South Islands since 1900, and some subspecies are threatened (Robertson et al., 2007), but weka can be a controversial species in New Zealand conservation because of their predatory impacts on native fauna, including birds (Harper, 2006), herpetofauna (Lettink, Hopkins, & Mayhew, 2010), and invertebrates (Gibbs, 2010). At times, this has resulted in their exclusion from mainland restoration projects, even in areas where they historically occurred (Miskelly & Beauchamp, 2004). Importantly, our study has highlighted the positive ecosystem services that they also provide, with weka dispersing the majority of hinau fruits from the ground on island sanctuaries. Given that the low fruit handling rates we recorded from the canopy suggested hinau is not regularly dispersed by volant birds, weka appear to be the primary disperser for hinau where they are present. This is concerning given that weka are now extinct over large tracts of their historic range (including most of the range of hinau) due to a combination of mammalian predation, habitat loss, and possible drought‐induced starvation (Miskelly & Beauchamp, 2004). Their susceptibility to rapid population declines makes conservation action even more pressing (Beauchamp et al., 1999).

In addition to hinau, weka have also been recorded consuming the fruits of a wide range of other plants, including *Geniostoma lingustrifolium* var. *rupestre*,* Coprosma* spp., *Passiflora tetranda*,* Piper excelsum*,* Pseudopanax arboreus*,* Prumnopitys ferruginea, Carpodetus serratus,* and *Pennantia corymbosa* (Beauchamp, 1987; Coleman et al., 1983). Several weka dietary studies have recorded weka eating large amounts of fruit in certain seasons (Beauchamp, 1987; Coleman et al., 1983), so the seed dispersal services these birds provide for other plant species could be considerable. However, effective seed dispersal includes both dispersal quantity (the number of seeds dispersed) and dispersal quality (the treatment of seeds in the mouth and gut, and the locations of seed deposition) (Schupp, Jordano, & Gómez, 2010). While weka provided good dispersal quantity for hinau, further research is required to assess the quality of dispersal they provide. Mechanical scarification of the seed coat has been shown to increase the germinability of hinau seeds (Carpenter et al., 2018), and it is possible that the grit within weka gizzards (Carroll, 1963) may abrade hinau seeds during gut passage in a similarly beneficial way. Germination trials using weka‐passed seeds from a wide range of plant species would be useful. Similarly, mechanistic models that combined both gut passage times and high‐resolution movement patterns for weka would further clarify their seed dispersal capabilities.

### 4.2 Importance of ground dispersal

Our findings strongly suggest that hinau fruits were primarily dispersed by flightless birds in prehuman New Zealand. We recorded high levels of fruit removal from the ground on islands that retain much of their prehuman avifauna, and fruit handling rates from the canopy on islands were still poor despite higher numbers of volant frugivores. While fruit handling rates do not provide information on quantitative seed dispersal (as successful seed dispersal typically requires the movement of fruits away from beneath parent tree canopies), fruit handling rates are monotonically related to the percentage of seeds that are moved away from beneath the parent canopy and therefore they are an index of dispersal quantity (Wyman, 2013). Our fruit handling rates are therefore probably lower than the actual dispersal rate, although they are likely to also include seeds that have been consumed at other hinau trees and dispersed away from the parent canopy. Although it is difficult to objectively define what constitutes “poor” fruit handling rates, the indices we recorded here are lower than those found for other New Zealand native fruiting trees (such as miro *Prumnopitys ferruginea*, matai *Prumnopitys taxifolia*, rimu *Dacrydium cupressinum*, and kahikatea *Dacrycarpus dacrydioides*; Carpenter et al., 2017) that are dispersed by smaller still widespread frugivores. In addition, we found very low avian visitation rates to hinau canopies, both on the mainland and on island sanctuaries, which suggests that hinau is not very attractive to volant dispersers.

While we recorded reasonably high levels of fruit removal from the ground on sanctuary islands, prehuman levels of ground fruit removal could have been even higher. There would have been far richer species diversity and greater abundances of flightless birds in New Zealand's prehuman ecosystems (potentially >27 spp.; Atkinson & Millener, 1991), and even if their diets were not primarily frugivorous, these flightless birds would have likely moved many seeds due to their sheer abundance (Lord, 2002). Furthermore, even volant frugivorous birds probably spent more time foraging on the ground prior to the arrival of mammalian predators (Wotton, 2007). Kereru, tieke, and kakariki are frequently seen feeding on the ground on islands that are free of mammalian predators (Innes et al., 2010; Wotton, 2007), and we recorded kereru consuming hinau fruits from the ground on Blumine Island.

This study is the first report of high levels of seed dispersal by a flightless bird in New Zealand. Cassowaries and emus are key seed dispersers for many plant species in Australia, consuming a wide variety of seeds and moving them large distances (e.g., Bradford & Westcott, 2010; Calviño‐cancela et al., 2006). Taken together, these results suggest that ground removal of fruit by flightless birds may be or have been an important dispersal mechanism in other parts of the world. Flightless birds are common on oceanic islands that lack mammalian predators, but such birds have frequently undergone severe declines or extinctions since human arrival. Duncan, Boyer, and Blackburn (2013) demonstrated that across the Pacific, flightless birds were 33 times more likely to have gone extinct than volant birds. For example, Hawai'i harbored at least 20 species of flightless birds before human arrival, including 12 rails (Olson & James, 1991). Greater Polynesia has also suffered from major losses of ground‐dwelling birds, with Steadman (1995) estimating that “flightless rails alone may account for 2,000 species of [extinct] birds that would have been alive today had people not colonized Oceania.” Dispersal may be reduced if these birds historically performed seed dispersal services, but their possible contributions are rarely examined or considered. Megapodes (*Megapodius* spp.), for example, may be significant seed dispersers, but this has never been investigated and many species from this genus are now extinct (Meehan, McConkey, & Drake, 2002).

### 4.3 Seed predation

Exotic rodents were the most common species to remove and presumably destroy seeds at mainland sites, while no seeds were destroyed on the ground on the islands. Similarly, Overdyck et al. (2013) recorded ~40% of hinau seed being removed by exotic rodents in an urban forest remnant after 3 weeks, and Daniel (1973) recorded ship rats destroying 21% of hinau seeds under parent trees. The rodents now in New Zealand (mice, ship rats, Norway rats, and Polynesian rats) do not display scatter‐hoarding behavior (i.e., burying seeds in widely spaced caches; Vander Wall, 1990), which is the typical mechanism of seed dispersal by rodents in the Northern Hemisphere. Instead, these taxa display caching behavior (Morriss, Warburton, Cross, & Nugent, 2012; Williams, Karl, Bannister, & Lee, 2000), where they carry seeds away for consumption at sites where they are safe from predators, competitors, and rain. Previous research suggests that the majority of cached hinau seeds end up destroyed. On Tiritiri Matangi Island, 62% of hinau seeds found in Polynesian rats’ “husking stations” were destroyed (Campbell, Moller, Ramsay, & Watt, 1984), while Beveridge (1964) recorded finding “piles of [rodent] gnawed miro and hinau seed … under logs and in other sheltered positions in the forest.” Mice have also been discovered caching hinau seeds in plastic tunnels that are intermittently used as bait stations, with most seeds destroyed (J. Ledington personal communication 2017). Because the seeds are not buried, and cache sites are typically sheltered, dark, dry places, uneaten seeds have little chance of establishing. For example, Polynesian rats cache seeds in tree roots, fissures in tree trunks, among rock piles, and occasionally up trees (Campbell et al., 1984). Similarly, exotic rats in Hawai'i moved a large proportion of palm seeds up to 8 m away from their collection site and subsequently destroyed them (Shiels & Drake, 2015).

We also assumed that the majority of seeds consumed by feral pigs were destroyed. Large quantities of destroyed hinau seeds have been reported in the guts of feral pigs (Beveridge, 1964), and O'Connor and Kelly (2012) found that feral pigs passed intact only 14% of New Zealand matai seeds (*Prumnopitys taxifolia*). Matai seeds are only slightly smaller than hinau with a similar hard, woody endocarp so we anticipate that the survival rates are probably similar. While we recorded only low numbers of pigs removing hinau seeds, this probably reflects low pig densities rather than dietary preferences. In New Zealand, feral pigs have a patchy distribution and can range widely to forage on preferred foods, so local pig densities vary greatly in space and time (King, 2005). Hinau seeds are a popular food choice for pigs, making up 30.9% of their diet in combination with tawa *Beilschmiedia tawa* at a North Island site (Thomson & Challies, 1988).

Endemic parrots (kaka and kakariki) destroyed 32.5% of seeds from the canopy at the island sites over an entire 6‐month fruiting season. Kaka are formidable seed predators and have been recorded destroying an average of 7.1 hinau seeds per minute on Kapiti Island (Moorhouse, 1997). In the early stages of human settlement, kaka were extremely abundant and the impact of their seed predation on favoured tree species was probably immense. Hinau's highly variable crops may therefore have evolved to satiate parrot seed predators during heavy fruiting years, enabling a proportion of the crop to survive (Kelly & Sork, 2002; Koenig et al., 2003). While the seed predation rates we recorded for endemic parrots appear higher than those recorded for exotic mammals on the mainland (21.5%), it is important to note that we cannot directly compare these two measures of seed predation as they use different monitoring methods (seed traps vs. camera footage) over different time spans (6 months for parrots vs. 2 weeks for mammals). However, it seems likely the proportion of seeds destroyed by exotic mammals would have increased if we had monitored over the entire season. For example, Overdyck et al. (Overdyck et al., 2013) observed that the proportion of hinau fruit removed by exotic mammals continued to increase over 50 weeks, although the rate of seed removal slowed after 3 weeks.

Finally, we recorded possums chewing large proportions of hinau seeds both on the ground and in the canopy at mainland sites. While these interactions did not destroy the hinau seeds, the removal of the carbohydrate‐rich mesocarp may make these seeds less attractive to legitimate dispersers. In addition, possums negatively affect hinau recruitment by consuming hinau flowers and significantly suppressing fruit production (Cowan & Waddington, 1990), so their impact on the tree is largely deleterious.

## 5 CONCLUSION

We found that ground‐based dispersal of hinau is impaired on the New Zealand mainland compared to sanctuary islands, due to low frugivore numbers. Seeds on the ground at mainland sites were most likely to be removed by exotic seed predators, while seeds on the ground on island sanctuaries were most likely to be removed by endemic seed dispersers. This study has also highlighted the importance of an unexpected disperser for hinau, the charismatic but controversial weka. This finding demonstrates the importance of testing which species perform important mutualistic services, rather than simply relying on logical assumptions. In future, conservation management decisions regarding the removal (or nonreintroduction) of weka in restoration projects should carefully consider the seed dispersal services they provide. Further research is needed to assess whether the reduced dispersal we observed is reducing recruitment.

## CONFLICT OF INTEREST

We have no competing interests to declare.

## AUTHOR CONTRIBUTIONS

JKC and DK conceived and designed the study. JKC conducted fieldwork with logistical assistance from COD. JKC and EM performed statistical analyses. JKC wrote the manuscript and DK, COD, and EM provided editorial advice.
